# Two decades of advances in preeclampsia research: molecular mechanisms and translational studies

**DOI:** 10.1172/JCI184052

**Published:** 2024-08-01

**Authors:** S. Ananth Karumanchi

**Affiliations:** Department of Medicine, Cedars-Sinai Medical Center, Los Angeles, California, USA.

Preeclampsia, a hypertensive disorder of pregnancy, poses a serious threat to the health of women and infants, causing over 75,000 maternal deaths and more than 500,000 infant deaths annually worldwide. Its grim toll is compounded by adverse maternal complications, such as seizures (eclampsia), liver dysfunction, or pulmonary edema. Although interventions like antihypertensives and magnesium sulfate offer partial mitigation of risks to the mother, the definitive remedy remains delivery of the infant and placenta. However, this solution begets iatrogenic prematurity, ushering infants into a neonatal period fraught with serious complications, such as bronchopulmonary dysplasia and necrotizing enterocolitis, as well as other long-term consequences.

In 1914, James Young proposed that interference with uterine blood supply to the placenta would lead to placental infarctions that, in turn, would release toxins into the maternal circulation, thus causing eclampsia (summarized in ref. 1). Decades later, during the mid-1980s, Roberts and Taylor hypothesized that preeclampsia is a endothelial disease based on the observation that the endothelium was the predominant cell type injured during preeclampsia and because endothelial injury antedated the clinical manifestations of disease (2). In a 2003 *JCI* paper, our group reported evidence and posited that elevated levels of soluble fms-like tyrosine kinase 1 (sFLT1) secreted from the placenta acted as the putative “endothelial toxin” that drives the clinical syndrome of preeclampsia (3). Below, we describe how these initial findings and subsequent papers (many published in the *JCI*) have transformed our understanding of preeclampsia and contributed to significant advances in the care of women with this disease.

## Early experiments suggesting placental sFLT1 causes maternal preeclampsia

I began my scientific career under the mentorship of senior physician-scientists, Frank Epstein and Vikas Sukhatme, at the Beth Israel Deaconess Medical Center, who encouraged me to pursue the molecular underpinnings of preeclampsia. We hypothesized that the systemic maternal endothelial injury in preeclampsia must originate in placenta, as the disease improves after delivery. Using transcriptional profiling of discarded placentas from normal patients and patients with preeclampsia, we screened for secreted proteins that could cause systemic endothelial injury in the maternal vasculature. To our delight and surprise, among the most upregulated mRNAs was a family of genes that belong to the VEGF receptor family, referred to as *sFLT1* (also known as *sVEGFR1*) (3). sFLT1 is an endogenous inhibitor of VEGF signaling that was first identified by Kendall et al. from Merck Pharmaceuticals; however, the biological role was not known (4). In our first *JCI* paper describing this pathophysiology, we reported that circulating sFLT1 levels are 3- to 5-fold higher in women with preeclampsia when compared with patients with normotensive pregnancies, and they fell after delivery of the placenta (2). Overexpression of *sFlt1* in pregnant rats was sufficient to induce hypertension, proteinuria, and glomerular endotheliosis, the classic renal histological lesion of preeclampsia. In clinical studies done in collaboration with Richard Levine at the NIH and Ravi Thadhani at the Massachusetts General Hospital, we then demonstrated that rises in circulating sFLT1 preceded the clinical onset of signs and symptoms of preeclampsia (5).

**Figure 1:**
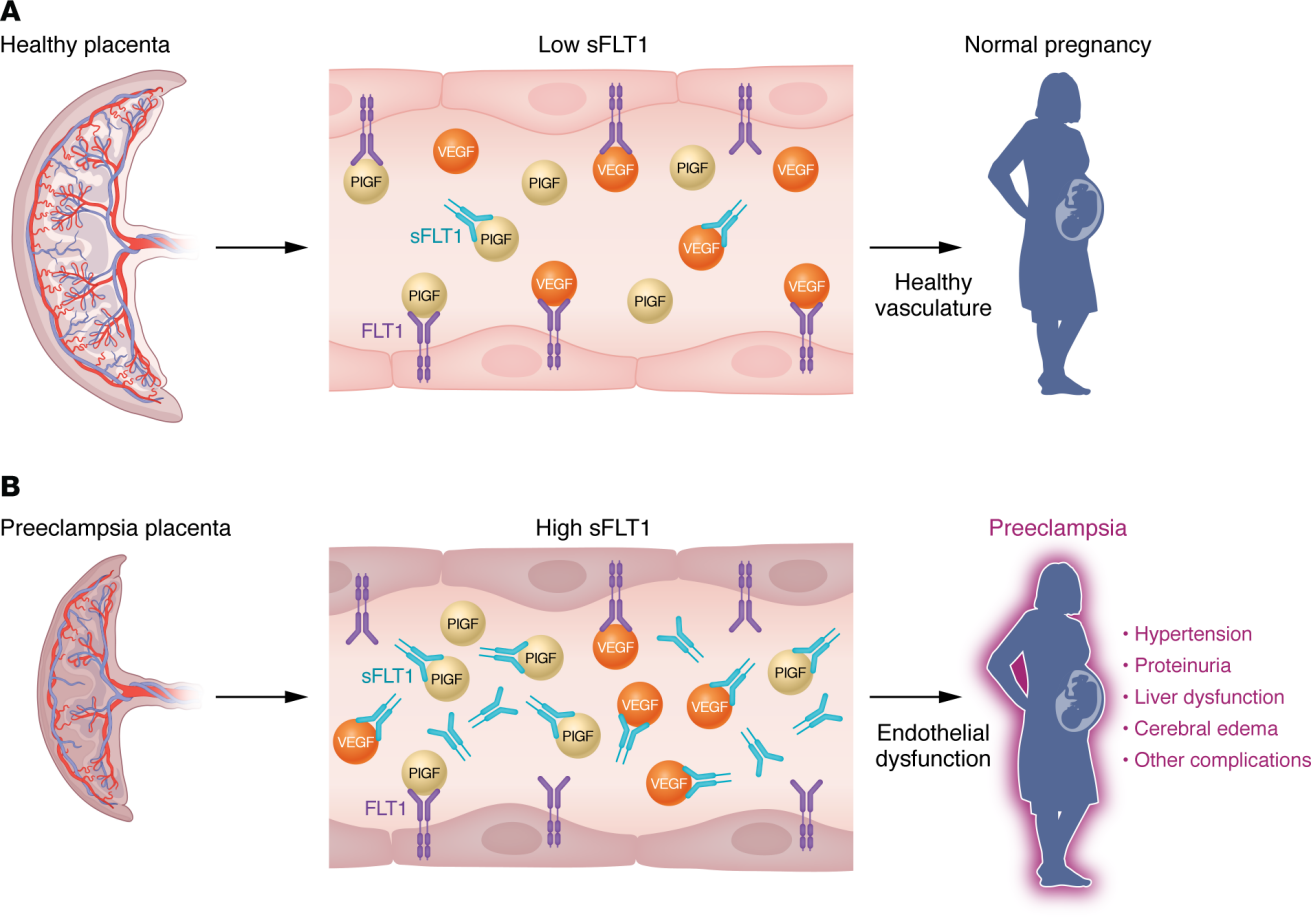
Schematic of the role of sFLT1 in mediating preeclampsia. (**A**) During normal pregnancy, the placenta produces modest concentrations of sFLT1 to balance the rising production of VEGF and PlGF, which in turn maintains vascular homeostasis. (**B**) In preeclampsia, excess placental production of sFLT1 binds VEGF and PlGF in the vasculature and prevents their interaction with endothelial cell–surface receptors. This results in endothelial cell dysfunction, including decreased prostacyclin and nitric oxide production as well as release of endothelin-1 and procoagulant proteins that, in turn, leads to the clinical syndrome of preeclampsia.

The maternal vascular beds that are affected in preeclampsia, such as the renal glomerular capillaries or hepatic sinusoidal endothelium, are largely characterized by fenestrated endothelial substructure to allow filtration of large macromolecules. Palade and others had previously demonstrated in cell culture studies that VEGF was critical for the maintenance of endothelial fenestrae (6), but in vivo evidence was lacking. Meanwhile, in an article appearing in the same March 2003 issue of *JCI*, Sue Quaggin’s laboratory at the University of Toronto published a mouse model study showing that a 50% reduction of VEGF production from the podocytes in kidney glomeruli led to loss of endothelial fenestrae and proteinuria, producing a state resembling preeclampsia (7). Taking these results together with our experimental and human studies, we then postulated that the maternal syndrome of preeclampsia was largely mediated by high levels of sFLT1 secreted from placental tissue (which is fetal in origin) acting on maternal vascular beds that were particularly dependent on constitutive VEGF signaling for the maintenance of endothelial fenestrae and health. Intriguingly, this pathophysiologic model was consistent with the evolutionary hypothesis first proposed in 1993 by David Haig, at Harvard University, that competing interests between the mother and fetus may underlie the development of preeclampsia (8). Indeed, subsequent large genome-wide association studies have revealed further corroborating evidence that fetal variants in the FLT1 locus are robustly associated with preeclampsia (9). In summary, experimental, genetic, and clinical evidence suggested that circulating sFLT1 of placental origin could be the link between placental disease and the maternal vascular endothelial dysfunction of preeclampsia (see Figure 1).

## Mechanistic studies of the basis of preeclampsia

Pregnancy is characterized by expansion of plasma volume due to upregulation of the renin-angiotensin system; however, during pregnancy the vasculature is generally resistant to the vasoconstrictive effects of angiotensin II. In 1973, Gant et al. from the University of Texas Southwestern Medical School published a seminal paper in the *JCI* in which they demonstrated that women who eventually developed preeclampsia first experienced angiotensin II sensitivity, followed by rising blood pressure and subsequent progression to preeclampsia (10). Since the timing of the enhanced angiotensin II sensitivity coincided with the rise in sFLT1 noted, we hypothesized that excess sFLT1 could directly contribute to angiotensin II sensitivity. In a subsequent *JCI* publication, we reported that sFLT1 mediated the enhanced angiotensin II sensitivity and hypertension noted in preeclampsia by inhibiting VEGF-dependent endothelial nitric oxide synthase and nitric oxide production (11).

In addition to observing increased concentrations of sFLT1 in preeclampsia, a parallel decrease was noted in detectable circulating concentrations of placental growth factor (PlGF), a second circulating member of the VEGF family. However, because PlGF does not bind to the important VEGF signaling receptor VEGFR-2 (also known as KDR), its role in preeclampsia was debated. In yet another *JCI* paper, Parchem et al. reported that genetic loss of PlGF in mice was not associated with hypertension and proteinuria during late gestation, suggesting that low PlGF concentrations were unlikely to contribute to the development of preeclampsia (12). Subsequently, we discovered that total PlGF levels were actually normal in women with preeclampsia, but the reason that PlGF levels appeared so low was because the PlGF antigen was being tightly bound by sFLT1, masking it from detection by immunoassay (13).

What are the upstream regulators of sFLT1 production in the placenta? Fan et al. provided evidence reported in the *JCI* that fetal-derived trophoblast cells overexpress sFLT1 in defense against excessive VEGF produced by the maternal decidual cells (14); however, this etiology is mostly restricted to specific subtypes of preeclampsia that may have decidual origins. Studies modeling pregnancy in women with a single kidney (who are at risk for preeclampsia) gave additional clues to the upstream regulators. In 2022, our group found that uninephrectomized mice developed late hypertension and proteinuria during pregnancy resembling preeclampsia and that the phenotypes were related to suppressed kynurenine (a tryptophan metabolite) production in the placenta that led to higher sFLT1 production (15). Intriguingly, kynurenine replacement led to normalization of sFLT1 and amelioration of preeclampsia signs and symptoms, suggesting it has a role in mediating the association between decreased renal reserve and placental distress. Susan Fisher’s laboratory previously provided evidence in the *JCI* that the gene expression changes noted in preeclampsia were reversible, suggesting that therapies aimed at reversing defective placentation and preventing preeclampsia are possible (16).

## Translational studies of sFLT1

Measurement of sFLT1 and its soluble binding partner PlGF has emerged as a diagnostic/prognostic tool in preeclampsia. Starting in early pregnancy, sFLT1 and PlGF concentrations rise in parallel with gestational age and growth of the placenta. As a result, assessment of whether concentrations of either sFLT1 or PlGF individually are within normal limits is difficult to interpret unless one knows the gestational age. However, since the sFLT1 and PlGF concentrations are normally proportional to each other and their ratio is relatively constant until term (~37 weeks) in a normal pregnancy, and because sFLT1 concentrations rise disproportionately during development of preeclampsia, measurement of the sFLT1/PlGF ratio becomes a highly predictive test to assess risk of developing preeclampsia and its related complications. In 2006, we published in the *New England Journal of Medicine* the ratio test to describe the temporal relationship between these biomarker levels and onset of clinical disease (17), and nearly a decade later, the sFLT1/PlGF ratio test was approved by the FDA and European regulatory authorities for risk stratification in women with suspected preeclampsia during the preterm gestational period (18, 19). The sFLT1/PlGF ratio test also predicts maternal and perinatal adverse events associated with preterm preeclampsia within two weeks of testing (18).

In addition to use of sFLT1 as a clinical biomarker for predicting preeclampsia development, clinical and experimental studies outlined above have strongly suggested that targeting sFLT1 and the VEGF signaling pathway may be a viable strategy to prevent or treat preeclampsia. In human studies led by my long-term collaborator, Ravi Thadhani at Massachusetts General Hospital, we demonstrated that a 30%–40% reduction in circulating sFLT1 levels using dextran sulfate apheresis is sufficient to ameliorate preeclamptic signs and symptoms and prolong pregnancy duration (20, 21). Proof-of-concept clinical trials with adsorption columns using sFLT1 antibody, which is more specific than dextran sulfate are ongoing (NCT02923206). Other strategies such as RNA interference strategies and small molecules that modulate sFLT1 production and/or actions are also being pursued to prevent or treat preeclampsia (22–24).

## Summary

Preeclampsia has fascinated clinicians and scientists since ancient times and was often referred to as the “disease of theories.” The discovery of the sFLT1 pathway in preeclampsia has led to molecular diagnostics that are helping obstetricians risk-stratify and manage patients appropriately (24). The test itself has the potential to save lives by allowing early detection of the condition. Looking ahead, the road map to conquering preeclampsia appears clearer than ever. Armed with molecular insights and burgeoning therapeutic modalities, we stand on the cusp of a new era in obstetric medicine.

## Version 1 08/01/2024

Electronic publication
